# Impact of recruitment strategies on individual participation practices in the Canadian National Vaccine Safety Network: prospective cohort study

**DOI:** 10.3389/fpubh.2024.1385426

**Published:** 2024-08-12

**Authors:** Phyumar Soe, Manish Sadarangani, Monika Naus, Matthew P. Muller, Otto G. Vanderkooi, James D. Kellner, Karina A. Top, Hubert Wong, Jennifer E. Isenor, Kimberly Marty, Gaston De Serres, Louis Valiquette, Allison McGeer, Julie A. Bettinger, for the Canadian Immunization Research Network

**Affiliations:** ^1^Vaccine Evaluation Center, BC Children’s Hospital Research Institute, Vancouver, BC, Canada; ^2^School of Population and Public Health, University of British Columbia, Vancouver, BC, Canada; ^3^Department of Pediatrics, University of British Columbia, Vancouver, BC, Canada; ^4^BC Center for Disease Control, Vancouver, BC, Canada; ^5^Department of Medicine, Unity Health Toronto, Toronto, ON, Canada; ^6^Department of Pediatrics and Alberta Children’s Hospital Research Institute, University of Calgary, Calgary, AB, Canada; ^7^Department of Pediatrics, Canadian Center for Vaccinology, IWK Health, Dalhousie University, Halifax, NS, Canada; ^8^Department of Pediatrics, Dalhousie University, Halifax, NS, Canada; ^9^College of Pharmacy, Dalhousie University, Halifax, NS, Canada; ^10^Department of Microbiology and Infectious Diseases, Université de Sherbrooke, Sherbrooke, QC, Canada; ^11^Department of Microbiology, Sinai Health System, Toronto, ON, Canada; ^12^Department of Laboratory Medicine and Pathobiology, University of Toronto, Toronto, ON, Canada

**Keywords:** COVID-19 vaccines, technology-assisted active recruitment, auto-invitation, auto-enrollment, vaccine safety

## Abstract

**Background:**

The Canadian National Vaccine Safety (CANVAS) network conducted a multi-center, prospective vaccine safety study to collect safety data after dose 1 and 2 of COVID-19 vaccines and follow up safety information 7 months after dose 1.

**Objective:**

This study aimed to describe and evaluate the recruitment methods used by CANVAS and the retention of participants by each modality.

**Methods:**

CANVAS deployed a multi-pronged recruitment approach to reach a larger sample, without in-person recruitment. Three primary recruitment strategies were used: passive recruitment, technology-assisted electronic invitation through the vaccine booking system (auto-invitation), or auto-registration through the vaccine registries (auto-enrollment).

**Results:**

Between December 2020 and April 2022, approximately 1.3 million vaccinated adults either self-enrolled or were auto-enrolled in CANVAS, representing about 5% of the vaccinated adult Canadian population. Approximately 1 million participants were auto-enrolled, 300,000 were recruited by auto-invitation, and 5,000 via passive recruitment. Overall survey completion rates for dose 1, dose 2 and the 7-month follow-up surveys were 51.7% (681,198 of 1,318,838), 54.3% (369,552 of 681,198), and 66.4% (452,076 of 681,198), respectively. Completion rates were lower among auto-enrolled participants compared to passively recruited or auto-invited participants who self-enrolled. However, auto-enrolled samples were much larger, which offset the lower completion rates.

**Conclusion:**

Our data suggest that auto-enrollment provided an opportunity to reach and retain a larger number of individuals in the study compared to other recruitment modalities.

## Introduction

Post-marketing vaccine safety surveillance plays an essential role in early detection and investigation of adverse events following immunization for an appropriate public health response and evidence-based immunization policy (1, 2). Such assessment can detect unintended effects due to a vaccine or the vaccination procedures, and support confidence in vaccination programs (1). The Canadian National Vaccine Safety (CANVAS) network, established in 2009, is an active, participant-centered surveillance system that provides volunteer participant-based reporting of health events following vaccination (3). Over its 14 years, CANVAS has proven instrumental in providing rapid safety information to public health authorities (4–8).

The SARS-CoV-2 virus, first identified in December 2019 in Wuhan City, China, quickly led to a global pandemic which was declared on March 11, 2020 (9). In response, an unprecedented global collaboration expedited the development, testing, and emergency authorization of multiple COVID-19 vaccines within a year (10–13). The Pfizer-BioNTech vaccine, the first to receive emergency use authorization in December 2020, was quickly followed by other COVID-19 vaccines around the world, including in Canada (12). Prior to the implementation of COVID-19 vaccines in Canada, CANVAS was prepared to monitor short and medium-term of COVID-19 vaccine safety, playing a vital role in monitoring the safety of Canada’s COVID-19 vaccine program (14–16).

During the COVID-19 pandemic, it was anticipated that participant recruitment for vaccine safety surveillance could be challenging due to the rapidity and unpredictability of vaccine rollouts, challenges with vaccine supply, changing vaccine recommendations, and limited bandwidth among public health partners and other stakeholders (17). The required social distancing (18) made more traditional recruitment methods, such as in-person recruitment, impractical. Prior to the pandemic, CANVAS recruited participants both in person from hospital vaccination clinics, pharmacies, physicians’ offices, public health clinics, and mass vaccination clinics, as well as by email invitation sent to previous CANVAS participants (6, 7). However, the COVID-19 pandemic required a shift from in-person to virtual recruitment strategies to contact and engage potential study participants.

In addition, larger and ideally representative samples across all age groups and for all COVID-19 vaccine products were required to identify potential safety signals. Thus, CANVAS deployed a multi-pronged recruitment approach to reach a large number of individuals without in-person recruitment. Populations of particular interest included people who were pregnant, immunocompromised, or older. Advances in technology have enabled the virtual recruitment of research participants, leading to in increased use of technology-assisted participant recruitment methods in research studies, including vaccine safety studies (19–21). However, there remains limited literature on participants’ response rate, completion, or retention rates in large vaccine safety studies. Specifically, data on long-term participant engagement are sparse, raising question about whether study samples accurately represent the target population. This paper describes and evaluates the recruitment methods used by CANVAS and the retention of participants by each modality. We hypothesized both survey completion rates and participants demographics would vary according to the recruitment method used.

## Methods

### Study design and setting

This was a multicenter, observational prospective cohort study. The network consisted of 7 study sites across Canadian provinces and territories: Alberta, British Columbia, Nova Scotia, Ontario, Prince Edward Island, Quebec, and Yukon. Research Ethics Board approval was obtained at all sites (British Columbia and Yukon: University of British Columbia Children’s & Women’s, Ref: H20-03704; Quebec: Centre Intégré univrsitaire de santé et de services sociaux de l’Estrie, Ref: MP-31-2021-4044; Nova Scotia and Prince Edward Island: Health Prince Edward Island and IWK Health Research, Ref: 1026400; Alberta: Conjoint Health REB, University of Calgary, Ref: REB20-2177; Ontario: Unity Health Toronto, Ref: 20–334). All participants provided informed consent electronically between December 2020 and April 2022.

### Study participants

Eligible participants were adults 15 years of age and older or parents/guardians of children 6 months to 14 years of age, with an active email address and telephone number, who could communicate in English or French and resided in one of the above seven Canadian provinces or territories. Individuals participated as controls (unvaccinated) and/or as vaccines depending on their COVID-19 vaccine status. Detailed study procedures have been previously described (16). No financial incentive was provided for participation.

For this analysis, we included vaccinated participants aged 20 years and above. Vaccinated participants were followed up to complete online questionnaires via email 8 days after vaccination with dose 1, 8 days after vaccination with dose 2, and 7 months after vaccination with dose 1. Participants who completed the dose 1 survey received the dose 2 and 7-month follow-up surveys.

### Recruitment strategies

In Canada, health care is coordinated at the provincial or territorial level and COVID-19 vaccine delivery differed by jurisdiction. Thus, recruitment strategies used varied by province and territory. Three primary recruitment strategies were used: (1) passive recruitment, (2) technology-assisted electronic invitation through the vaccine booking system (auto-invitation), and (3) technology-assisted auto-registration from the vaccine registries (auto-enrollment).

#### Passive recruitment

Passive recruitment involved researchers at different study sites publicizing study information to potential participants and allowing prospective participants to self-enroll (22). All study sites except in Quebec used a passive recruitment approach. Study information was distributed via posters, information cards, or pamphlets at vaccination clinics as well as promotion campaigns through local mass media (e.g., newspaper, radio, and television) and social media platforms, such as Facebook, Twitter, and Instagram. Interested participants were directed to visit the CANVAS website and self-register for the study. A quick response code that linked to the CANVAS registration page for each province/territory was included in all information and interested participants self-enrolled on the registration page. Site investigators also promoted the study with key stakeholders, policy makers, and journalists to inform the public about the study and provide the study website for interested participants.

#### Technology-assisted active recruitment

Technology-assisted active recruitment was done in two ways: electronic invitation through the vaccine booking system (auto-invitation) and auto-registration from the contact information in the vaccine registries (auto-enrollment).

The auto-invitation approach also required potential participants to visit the CANVAS website and self-register. However, they received direct solicitation about the study by email from provincial public health or a provincial vaccine booking system. Study sites in Alberta, Nova Scotia, and Ontario employed this approach. In Alberta and Ontario all individuals were first asked, either when they made their vaccine appointment or at the time of vaccination, about their willingness to receive emails related to vaccine research. Those who agreed to contact were sent an invitation email from the public health system with a link to the CANVAS website for the individual to then self-register. In Nova Scotia, all vaccinated individuals were invited to participate via an email from the provincial vaccine booking system with a link to the CANVAS website to self-register.

Auto-enrollment through vaccination registries did not require potential participants to visit the CANVAS website and self-register to be enrolled. This approach involved automatically enrolling all COVID-19 vaccinated individuals with their vaccine information from the vaccine registries and then sending an email study invitation with a link to the consent form and survey. The British Columbia and Quebec study sites employed an auto-enrollment approach. For both passive and auto-invitation approaches, enrollment was initiated by participants who self-registered and provided their contact and vaccine information to the study. In the auto-enrollment approach, barriers to enrollment were removed as minimal contact information and vaccine information was provided to the study and the potential participant was able to click on a link directly to the consent and survey.

### Study variables

This study utilized recruitment method, age group, sex and gender variables for analysis from dose 1, dose 2 and follow-up surveys. Participants’ age was collected in six groups: 20–29, 30–39, 40–49, 50–64, 65–79, and ≥ 80 years. Minimal demographic variables were collected to reduce participant burden.

### Statistical analysis

Complete cases analysis was conducted for all analyses as no variable had more than 5% missing data. Participant enrollment was calculated among the Canadian population who received at least 1 dose of a COVID-19 vaccine in participating provinces and territories. To calculate overall participant enrollment, the vaccinated adult population estimates (18 years and above) from the Government of Canada, as of December 2021, were used as denominators (23). The proportion of participant enrollment in CANVAS may be underestimated because our adult vaccinated participants were aged 20 and above (numerator) whereas vaccinated population estimates were only available for individuals 18 years and above (denominator).

We calculated survey completion rates stratified by different recruitment methods. The completion rate for the dose-1 survey was calculated using the number of participants enrolled by each method as the denominator and those who completed the dose-1 survey as the numerator. For the dose 2 and 7-month follow-up surveys, survey completion rates were calculated using the number of participants who completed the dose 1 survey as the denominator and the number of participants who completed the dose 2 and 7-month follow-up surveys as numerators as the dose 2 and follow up surveys were only sent to those who completed the dose 1 survey. A chi-square test was applied to test the differences between the recruitment methods and demographics (age groups, sex, and gender). Data analysis was completed in R software version 4.1.3 (R Foundation for Statistical Computing, Vienna, Austria) (24).

## Results

### Participants’ enrollment

**Figure 1:**
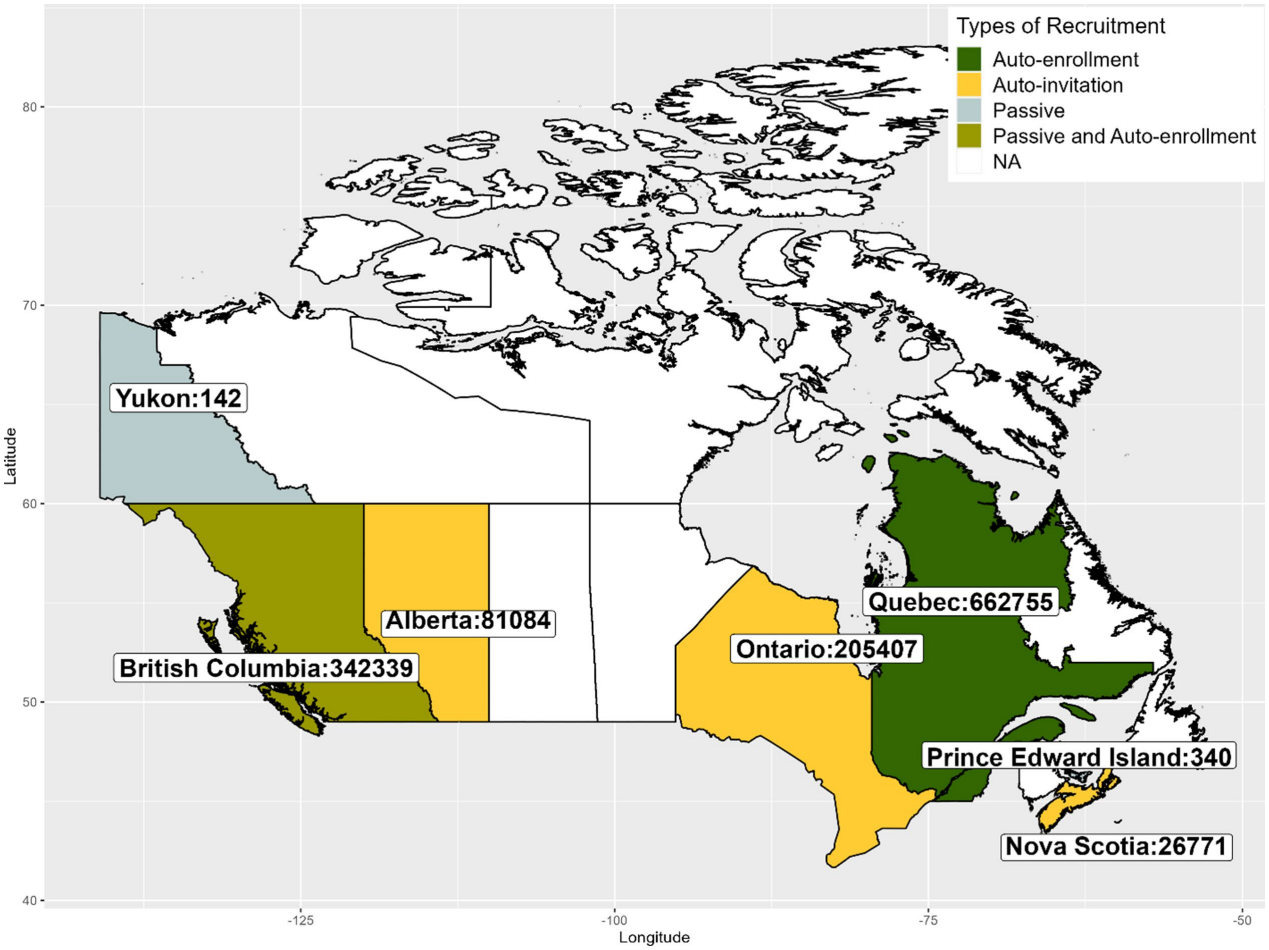
Number of individuals enrolled in CANVAS by Canadian Province/Territory between December 2020 and April 2022.

Between December 2020 and April 2022, approximately 1.3 million vaccinated adults enrolled, representing 4.7% of the adult Canadian population who received at least 1 dose of a COVID-19 vaccine. Of which, almost all enrolled via technology-assisted active recruitment (vaccination registries: 1,000,753 [75.9%] and vaccine booking system: 313,262 [23.8%]). About 5,000 participants (0.4%) enrolled through the passive recruitment method. Absolute numbers of enrollment by province/territory are presented in Figure 1.

### Participants’ survey completion rates by recruitment strategy

**Figure 2:**
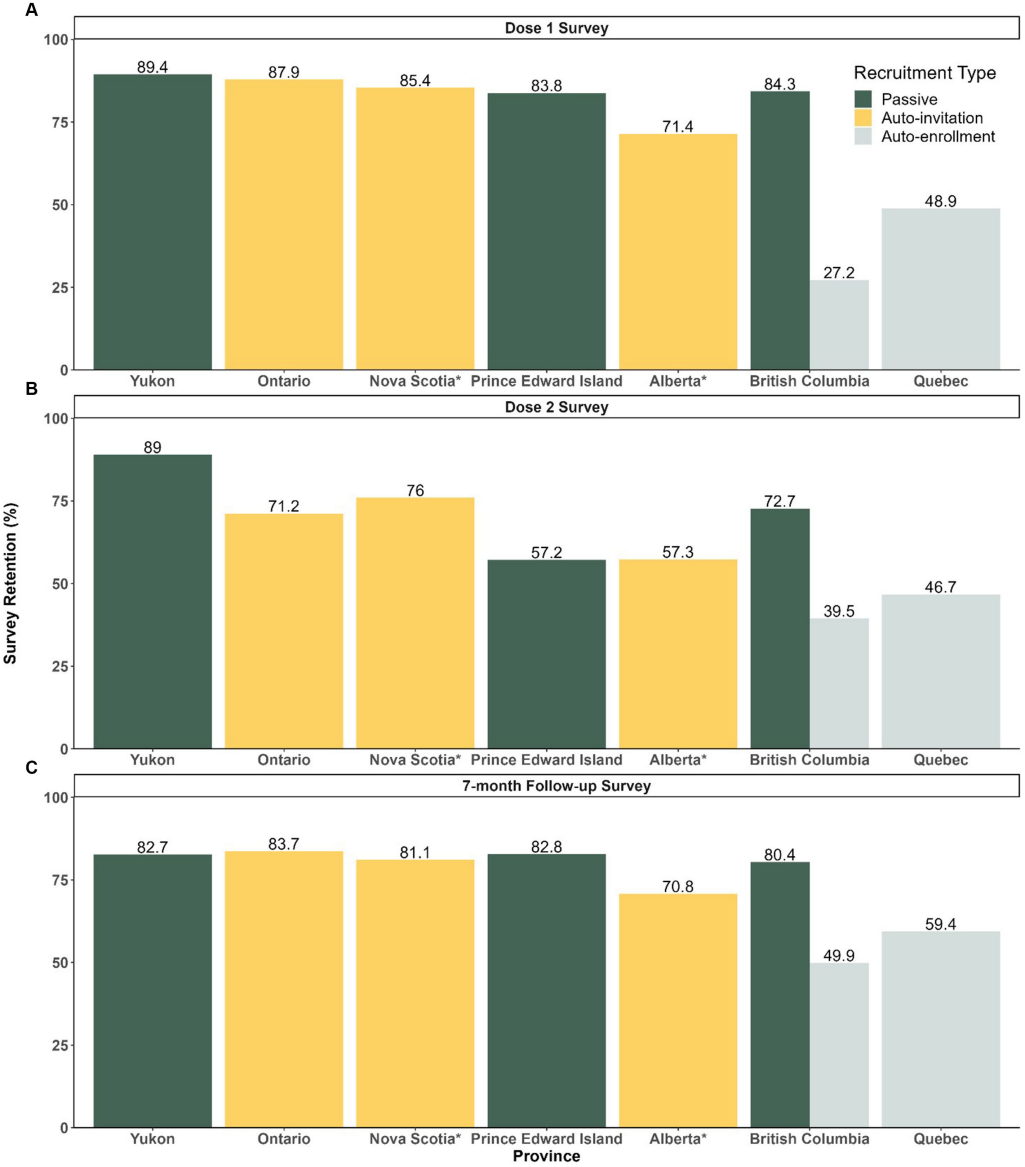
Survey Completion Rates among Participants by Recruitment Strategy and Jurisdiction for Dose 1, 2 and 7-month Follow-up Surveys **(A)** Dose 1 survey completion rates (number of individuals who completed dose 1 survey/ number of enrolled). **(B)** Dose 2 survey completion rates (number of individual who completed dose 2/number of individuals who completed dose 1). **(C)** Follow-up survey completion rates (number of individuals who completed follow-up/number of individuals who completed dose 1). *No data breakdown available between passive and vaccine booking systems for Alberta and Nova Scotia.

Out of 1.3 million vaccinated participants enrolled, overall survey completion rates for dose 1, dose 2 and 7-month follow-up surveys were 51.7, 54.3, and 66.4%, respectively. In general, the completion rates among participants recruited via passive recruitment method (dose 1: 4070/4823 [84.4%], dose 2: 2934/4070 [72.1%], and follow-up: 3281/4070 [80.6%]) and auto-invited participants (dose 1: 261332/313262 [83.4%], dose 2: 179099/261332 [68.5%], and follow-up: 210744/261332 [80.6%]) were higher than participants recruited via auto-enrollment (dose 1: 415796/1000753 [41.5%], dose 2: 187519/415796 [45.1%], and follow-up: 238051/415796 [57.3%]) (Figure 2).

### Participants’ demographics by recruitment strategy

Enrolling a sample with specific demographic characteristics was possible with auto-enrollment. For example, older age groups were targeted for the auto-enrolled samples, and older adults formed a larger proportion of respondents recruited via auto-enrollment than via other recruitment methods (Dose 1 65–79 years: auto-enrolled 26.5% Vs. passive/auto-invitation 19.0%, Dose 1 ≥ 80 years: auto-enrolled 4.1% *Vs* passive/auto-invitation 2.4%) (Table 1).

**Table 1:** Demographics (age, sex, and gender) among participants by recruitment strategy for dose 1, 2 and follow-up surveys.

Dose 1 survey					
Variable	Overall, *N* = 681,198^1^	Auto-enrollment, *N* = 415,796^1^	Auto-invitation, *N* = 261,332^1^	Passive, *N* = 4,070^1^	*p*-value^2^
Age groups					<0.001
20–29	72,795 (10.7%)	39,503 (9.5%)	32,909 (12.6%)	383 (9.4%)	
30–39	111,081 (16.3%)	58,901 (14.2%)	51,334 (19.6%)	846 (20.8%)	
40–49	104,436 (15.3%)	58,130 (14.0%)	45,599 (17.4%)	707 (17.4%)	
50–64	208,852 (30.7%)	131,987 (31.7%)	75,445 (28.9%)	1,420 (34.9%)	
65–79	160,696 (23.6%)	110,209 (26.5%)	49,815 (19.1%)	672 (16.5%)	
80 and above	23,338 (3.4%)	17,066 (4.1%)	6,230 (2.4%)	42 (1.0%)	
Sex					<0.001
Male	289,834 (42.5%)	185,319 (44.6%)	103,354 (39.5%)	1,161 (28.5%)	
Female	390,183 (57.3%)	229,591 (55.2%)	157,693 (60.3%)	2,899 (71.2%)	
Decline/other	1,181 (0.2%)	886 (0.2%)	285 (0.1%)	10 (0.2%)	
Gender					<0.001
Man	288,182 (42.3%)	184,258 (44.3%)	102,775 (39.3%)	1,149 (28.2%)	
Woman	386,861 (56.8%)	227,761 (54.8%)	156,240 (59.8%)	2,860 (70.3%)	
Two spirit*	3,419 (0.5%)	1,749 (0.4%)	1,627 (0.6%)	43 (1.1%)	
Other/decline	2,736 (0.4%)	2,028 (0.5%)	690 (0.3%)	18 (0.4%)	

Dose 2 survey					
Variable	Overall, *N* = 369,552^1^	Auto-enrollment, *N* = 187,519^1^	Auto-invitation, *N* = 179,099^1^	Passive, *N* = 2,934^1^	*p*-value^2^
Age groups					<0.001
20–29	31,344 (8.5%)	12,831 (6.8%)	18,276 (10.2%)	237 (8.1%)	
30–39	55,194 (14.9%)	22,201 (11.8%)	32,395 (18.1%)	598 (20.4%)	
40–49	50,713 (13.7%)	20,871 (11.1%)	29,358 (16.4%)	484 (16.5%)	
50–64	120,368 (32.6%)	64,550 (34.4%)	54,753 (30.6%)	1,065 (36.3%)	
65–79	98,927 (26.8%)	58,815 (31.4%)	39,591 (22.1%)	521 (17.8%)	
80 and above	13,006 (3.5%)	8,251 (4.4%)	4,726 (2.6%)	29 (1.0%)	
Sex					<0.001
Male	150,131 (40.6%)	81,179 (43.3%)	68,146 (38.0%)	806 (27.5%)	
Female	218,983 (59.3%)	106,074 (56.6%)	110,788 (61.9%)	2,121 (72.3%)	
Decline/other	438 (0.1%)	266 (0.1%)	165 (0.1%)	7 (0.2%)	
Gender					<0.001
Man	149,432 (40.4%)	80,846 (43.1%)	67,783 (37.8%)	803 (27.4%)	
Woman	217,336 (58.8%)	105,401 (56.2%)	109,845 (61.3%)	2,090 (71.2%)	
Two spirit*	1,694 (0.5%)	611 (0.3%)	1,054 (0.6%)	29 (1.0%)	
Other/decline	1,090 (0.3%)	661 (0.4%)	417 (0.2%)	12 (0.4%)	

7 month follow-up survey					
Variable	Overall, *N* = 452,076^1^	Auto-enrollment, *N* = 238,051^1^	Auto-invitation, *N* = 210,744^1^	Passive, *N* = 3,281^1^	*p*-value^2^
Age groups					<0.001
20–29	37,028 (8.2%)	14,741 (6.2%)	22,038 (10.5%)	249 (7.6%)	
30–39	66,657 (14.7%)	27,829 (11.7%)	38,168 (18.1%)	660 (20.1%)	
40–49	65,500 (14.5%)	29,329 (12.3%)	35,622 (16.9%)	549 (16.7%)	
50–64	148,002 (32.7%)	82,192 (34.5%)	64,601 (30.7%)	1,209 (36.8%)	
65–79	119,725 (26.5%)	74,186 (31.2%)	44,962 (21.3%)	577 (17.6%)	
80 and above	15,164 (3.4%)	9,774 (4.1%)	5,353 (2.5%)	37 (1.1%)	
Sex					<0.001
Male	187,471 (41.5%)	104,301 (43.8%)	82,252 (39.0%)	918 (28.0%)	
Female	264,084 (58.4%)	133,430 (56.1%)	128,298 (60.9%)	2,356 (71.8%)	
Decline/other	521 (0.1%)	320 (0.1%)	194 (0.1%)	7 (0.2%)	
Gender					<0.001
Man	186,605 (41.3%)	103,859 (43.6%)	81,832 (38.8%)	914 (27.9%)	
Woman	262,075 (58.0%)	132,548 (55.7%)	127,202 (60.4%)	2,325 (70.9%)	
Two spirit*	2,103 (0.5%)	845 (0.4%)	1,227 (0.6%)	31 (0.9%)	
Other/decline	1,293 (0.3%)	799 (0.3%)	483 (0.2%)	11 (0.3%)	

1 n (%).

2 Pearson’s Chi-squared test; Two spirt*: Two-spirit or binary; Tests are significant with or without “other or two-sprit groups” in sex and gender variables.

Auto-enrollment: British Columbia and Quebec, Auto-invitation: Alberta, Nova Scotia, Ontario, Passive: British Columbia, Prince Edward Island and Yukon; No data breakdown available between passive and vaccine booking systems for Alberta and Nova Scotia.

Unequal representation of sex and gender by recruitment method was observed across all surveys. In general, females were more likely to participate and complete all surveys (≥ 57%), and these sex differences varied significantly depending on the recruitment strategy (*p*-value <0.001). The greatest disparities existed in participants recruited via passive recruitment methods, followed by the participants recruited via auto-invitation (Table 1). Regarding gender, a similar over representation of women was observed; more than 57% of samples were women across all surveys and there was a significant difference by recruitment strategy (Table 1). Women participants comprised the largest proportion of enrollees recruited via passive strategy, followed by auto-invitation and auto-enrollment. Participants who identified themselves as two spirit (indigenous peoples in the lesbian, gay, bisexual, transgender, and queer (LGBTQ) community) (25), non-binary or other were less than ≤1% of the study population with slight variations across the provinces and by recruitment strategies.

## Discussion

CANVAS successfully enrolled a very large number of adult Canadians using a multi-pronged recruitment approach. Importantly, overall survey completion and retention within the study were quite high. Survey completion rates and demographics of the sample varied by recruitment strategy, indicating the type of strategy is important. Not surprisingly, survey completion rates were lower among auto-enrolled participants compared to self-registered (passive or auto-invited) participants. However, the auto-enrolled samples were much larger, which offset the lower completion rates, and included a higher proportion of male participants and men. Although both technology-assisted recruitment strategies (auto-invitation and auto-enrollment) provide an opportunity to reach more participants, the auto-enrollment method reduced barriers to participation and reached more people. Thus, participants recruited from this strategy represented more than 75% of the total participants, which is particularly important in vaccine safety studies where recruiting a larger sample is required to detect rare events (2).

One of the advantages of auto-enrolling participants directly from centralized vaccine registries is data quality (26, 27). We could rely on the accuracy, validity, cohesiveness, and timeliness of vaccine data to assess participants’ information such as vaccine product, lot number, and date of vaccine administration. Another superiority of auto-enrollment is the ability to apply targeted sample adjustments during the enrollment phase to ensure adequate representation of various age groups and vaccine products (19). Very few participants aged 80 years and above were recruited passively, and we reached most of our very senior participants via auto-enrollment. One reason for this may be the ability of this type of recruitment to reduce barriers to enrollment for this participant group. This finding is in line with previous evidence that better logistics and design to lower participant burden facilitates participant enrollment and retention (28, 29). Importantly, our findings confirm that members of this hard-to-reach population were indeed willing and able to participate in online research if they were provided full study characteristics in a low-barrier manner (30, 31).

Similar to other online survey research and previous CANVAS studies, our samples had more females than males participate (4, 7, 32). A systematic review on sex and gender reporting in COVID-19 studies reported that 71% (20 out of 28) of observational had more women than men in their sample compared to 24% (10 out of 41) in interventional studies (33). However, we found that samples recruited by auto-enrollment reduced the sex and gender imbalance compared to samples recruited by other methods.

In our study, we are unable to estimate response rates from passive recruitment and auto-invitation as we did not have the total number who were invited via these methods. Although an attempt was made to compare our study completion rate from auto-enrollment with response rates from other studies, this should be interpreted with caution. Shim et al. used a short messaging service invitation with the survey link to assess the immunogenicity of different COVID-19 vaccines, using information from the electronic health record (19). The response rate for that survey (46.5%) was similar to our auto-enrollment completion survey rates for the dose 1 survey. Another study (21), that deployed a multi-pronged recruitment method in the United States revealed the highest response rate from phone calls (13%), followed by email (11.9%), text messages (11.4%), and patient portal messages (9.4%). These findings were much lower than CANVAS participants’ completion rate for auto-enrolled vaccinated recipients. However, the response rates are not directly comparable as our study may have utilized different approaches to measure response or survey completion rates than the above-mentioned studies.

Our findings indicate that survey completion rates between auto-invitation and passive recruitment were comparable. The auto-invitation method utilized established trusted sources (vaccine booking systems) and sent out invitations (study sites: Alberta and Ontario) only to individuals who were interested in and agreed to participate in vaccine safety studies, this likely would reach a similar group as those who self-registered after hearing about the study via poster or other methods. However, the auto-enrollment method automatically enrolled all potential participants and invited them to participate. In fact, this process removed a barrier to participation and followed the Assume, Seek, and Know (ASK) Approach (31) to enhance clinical trial participation. We assumed all potential eligible vaccinated participants would be interested to know about our study, we provided study information, and explicitly asked whether they wanted to participate in our study and we made it easy for them to participate. This process may reduce disparities in communication and study offers by increasing the awareness of our study to all eligible participants. This kind of active recruitment is usually operationalized by researchers identifying targeted populations, groups, or residents of defined areas and recruiting participants from the known subject pools such as a mailing list or a clinical roster (22). Our findings provide evidence that this process led to a larger sample and is worth considering in other settings, particularly when a more diverse or representative sample is desired.

In our study, the auto-invitation method used in Alberta and Ontario did not follow an Assume, Seek, and Know (ASK) recruitment approach (31). All eligible participants who used the vaccine booking systems did not have a chance to seek and know the aim and detailed procedure of the study. Instead, interested participants had to first agree to be contacted about research before they were told about it. Although several factors can influence an individual’s decision to take part in a study, communication, and delivery of study information are paramount (34–36). It is important to note our inability to provide study information to all potential participants was due to the varying requirements of local research ethics boards (or provincial/territorial public health requirements). In restricting access to potential research participants, research ethics boards and other authorities should weigh the risk of the proposed research against the risk of excluding important and understudied groups in research. Our results demonstrate the Assume, Seek, and Know (ASK) auto-enrollment approach provided a larger and more gender and sex-balanced sample.

### Strengths and limitations

CANVAS’s response to COVID-19 vaccine safety surveillance was possible because of a robust vaccine safety monitoring system already in place with considerable vaccine safety experience and expertise. Established partnerships with public health authorities has enabled CANVAS to perform successful online recruitments via different digital technology platforms. In addition, organizational and credential support from authorities has enhanced people’s confidence and trust that participation and retention in research are safe, ethical, and benefiting the society. A variety of media campaigns, including print media, local and social media, and interviews with study teams, may have helped to increase public awareness and understanding about the purpose of study. Having media coverage may have been influential not only in recruitment but also in survey completion and retention rates.

However, our study findings have several limitations. Participating in CANVAS was voluntary and such participants may differ from the general population. Although self-selection is presumed to occur in all samples, it may be more likely to occur in self-registered (passive/auto-invited) samples as they expressed their interest and commitment toward the research. Self-selection bias is less likely to be operating extensively in auto-enrolled samples because this approach facilitated the lowest barrier to participation.

Regardless of the recruitment method, the dose 2 survey completion rate was lower than that of the 7-month follow-up survey. This is likely due to constraints in vaccine supply and distribution capacity, which resulted in delays in providing the second dose of the two-dose primary vaccine series in Canada and made the timing of the 2nd dose survey within 7 days of vaccination difficult (37). Practically this meant the delivery of the dose 2 survey could not be accurately timed with delivery of the second dose of vaccine and participants were only eligible to complete the survey within the first 7 days following dose 2 vaccination. This may explain the higher completion rate in the 7-month survey compared to the dose 2 survey.

Another limitation of our study is that not all provinces and territories utilized every recruitment method, and the study did not collect additional individual factors that may influence survey engagement. This restricts our ability to fully understand the factors contributing to retention rates across different provinces and territories. Although we defined our recruitment strategies based on barriers to participation, differences in recruitment may exist between provinces with similar recruitment approaches. Finally, our results may limit generalizability as COVID-19 vaccine rollout during the pandemic would be different from current COVID-19 campaigns across Canadian provinces and territories.

### Implications and future directions

We demonstrated technology-assisted recruitment, such as auto-invitation and auto-enrollment, overcame some limitations of traditional recruitment methods and demonstrated the ability to recruit and retain larger samples. Compared to auto-invitation, auto-enrollment was superior to reaching a much larger number of potential participants by providing detailed study information and allowing them to decide if the studies fit their needs, interest, and comfort level.

This auto-enrollment strategy offers great potential not only for future vaccine safety studies but also across other research domains. It enables a stratified selection of participants based on diverse demographic characteristics such as age, gender, ethnicity, and vaccine type. Further research is crucial to investigate the factors influencing participant engagement among the samples recruited with technology-assisted recruitment methods. Additionally, future studies should focus on optimizing these methods to further enhance participant engagement, potentially increasing the diversity and representativeness of study samples, which is essential for generalizing findings across different populations.

## Conclusion

CANVAS successfully enrolled around 5% of adult vaccinated Canadians. Although passive recruitment and auto-invitation were associated with higher survey completion and retention, auto-enrollment reached more individuals with more equal representation of sex and gender, so ultimately would be a preferred recruitment method.

## Data availability statement

The datasets presented in this article are not readily available because we do not have permission from CANVAS participants to share the data used in our study. Requests to access the datasets should be directed to the corresponding author.

## Ethics statement

The studies involving humans were approved by UBC Children’s & Women’s, CIUSSS de l’Estrie – CHUS, Health PEI, Conjoint Health Research Ethics Board, University of Calgary and Alberta Health Services, IWK Health, Unity Health Toronto, and CHU de Québec-Université Laval. The studies were conducted in accordance with the local legislation and institutional requirements. The participants provided their written informed consent to participate in this study.

## Author contributions

PS: Data curation, Formal analysis, Investigation, Methodology, Visualization, Writing – original draft, Writing – review & editing. MS: Conceptualization, Funding acquisition, Investigation, Methodology, Supervision, Writing – review & editing, Validation. MN: Investigation, Methodology, Supervision, Writing – review & editing. MM: Conceptualization, Funding acquisition, Investigation, Methodology, Project administration, Writing – review & editing. OV: Conceptualization, Funding acquisition, Investigation, Methodology, Project administration, Writing – review & editing. JK: Conceptualization, Funding acquisition, Investigation, Methodology, Project administration, Writing – review & editing. KT: Conceptualization, Funding acquisition, Investigation, Methodology, Project administration, Writing – review & editing. HW: Data curation, Formal analysis, Supervision, Validation, Writing – review & editing, Visualization. JI: Conceptualization, Funding acquisition, Investigation, Methodology, Project administration, Writing – review & editing. KM: Conceptualization, Data curation, Investigation, Project administration, Resources, Software, Validation, Writing – review & editing. GS: Conceptualization, Funding acquisition, Investigation, Methodology, Project administration, Resources, Writing – review & editing. LV: Conceptualization, Funding acquisition, Investigation, Methodology, Project administration, Writing – review & editing. AM: Conceptualization, Funding acquisition, Investigation, Methodology, Project administration, Writing – review & editing. JB: Conceptualization, Funding acquisition, Investigation, Methodology, Project administration, Resources, Supervision, Validation, Writing – review & editing.
